# Pregnancy planning and neonatal outcome - a retrospective cohort study

**DOI:** 10.1186/s12884-024-06401-6

**Published:** 2024-03-16

**Authors:** Isa Reuterwall, Jenny Niemeyer Hultstrand, Alisa Carlander, Maria Jonsson, Tanja Tydén, Merit Kullinger

**Affiliations:** 1 Department of Obstetrics and Gynecology, Region Västmanland, Västerås, Sweden; 2 Department of Women´s and Children’s Health, Uppsala University, Uppsala, Sweden; 3 Centre for Clinical Research Västmanland Hospital, Västeras, Sweden

**Keywords:** Neonatal outcome, Preterm birth, Small for gestational age, Sweden, Unplanned pregnancy

## Abstract

**Background:**

Unplanned pregnancy is common, and although some research indicates adverse outcomes for the neonate, such as death, low birth weight, and preterm birth, results are inconsistent. The purpose of the present study was to investigate associated neonatal outcomes of an unplanned pregnancy in a Swedish setting.

**Methods:**

We conducted a retrospective cohort study in which data from 2953 women were retrieved from the Swedish Pregnancy Planning Study, covering ten Swedish counties from September 2012 through July 2013. Pregnancy intention was measured using the London Measurement of Unplanned Pregnancy. Women with unplanned pregnancies and pregnancies of ambivalent intention were combined and referred to as unplanned. Data on neonatal outcomes: small for gestational age, low birth weight, preterm birth, Apgar score < 7 at 5 min, and severe adverse neonatal outcome defined as death or need for resuscitation at birth, were retrieved from the Swedish Medical Birth Register.

**Results:**

The prevalence of unplanned pregnancies was 30.4%. Compared with women who had planned pregnancies, those with unplanned pregnancies were more likely to give birth to neonates small for gestational age: 3.6% vs. 1.7% (aOR 2.1, 95% CI 1.2–3.7). There were no significant differences in preterm birth, Apgar score < 7 at 5 min, or severe adverse neonatal outcome.

**Conclusions:**

In a Swedish setting, an unplanned pregnancy might increase the risk for birth of an infant small for gestational age.

**Supplementary Information:**

The online version contains supplementary material available at 10.1186/s12884-024-06401-6.

## Introduction

Unplanned pregnancies are common worldwide with a great geographical variation. In 2012, 40% of all pregnancies worldwide were considered unplanned, and 38% ended in a live birth [1–4]. In 2015, 19% of pregnancies in Europe were reported as unplanned [5]. According to a study from Sweden conducted in 2012–2013, 12% of pregnancies ending in childbirth were fairly or very unplanned [6].

Compared with women who have planned pregnancies, those with unplanned pregnancies are more likely to be younger, multiparous, smokers, immigrants, have lower educational levels, and suffer from psychiatric illness, domestic violence, or both [3, 5–12]; all of which can constitute an increased risk of poor pregnancy outcomes.

Previous studies of unplanned pregnancies have had methodological limitations regarding the measurement of pregnancy planning. Mostly, pregnancy intention has been assessed retrospectively (up to five years after delivery) and was often based on a single question “was the pregnancy planned or unplanned?” Few studies have measured the intention before pregnancy [13, 14]. The London Measure of Unplanned Pregnancy (LMUP) is a validated scale that was developed to measure pregnancy planning. The LMUP is recommended to increase comparability between studies and is increasingly used internationally [4, 15, 16].

Studies about the associations between unplanned pregnancies and neonatal outcome are limited, and results are inconsistent [13, 14]. While, in some studies, unplanned pregnancy has been associated with low birth weight (LBW), neonates small for gestational age (SGA), preterm birth [17–20], neonatal mortality, and stillbirth [14, 15], no associations with neonatal outcome were found by other studies [3, 7, 9, 21–23]. The causal pathway between unplanned pregnancy and adverse neonatal outcome is unclear.

The birth weight of neonates can be reported either unrelated to gestational age, such as LBW, or related to gestational age, such as SGA and large for gestational age (LGA). SGA has a higher prevalence and is considered more clinically relevant than LBW. However, LBW is often used instead of SGA in settings without early or mid-pregnancy gestational dating with ultrasound. Both LBW and SGA are more common among preterm neonates. Preterm neonates, as well as SGA neonates, are in general at higher risk of perinatal morbidity and mortality, and globally, these complications are among the most common causes of perinatal death and can also lead to both physical and mental complications later in life [24–28]. Early antenatal care including early pregnancy dating give the possibility to detect SGA fetuses at subsequent ultrasound scans. This is important because antenatal detection reduces the risk for adverse pregnancy outcomes in SGA fetuses compared with those undetected before birth [29].

To our knowledge, there are no previous studies on the association between pregnancy planning and neonatal outcomes in Sweden. Further, only a few studies have so far used a validated tool to assess pregnancy planning to study outcomes related to an unplanned pregnancy.

## Aim

The aim of the present study was to investigate the association between unplanned pregnancies and adverse neonatal outcomes, including perinatal death, resuscitation measures, SGA, LBW, preterm birth, and low Apgar score.

## Method

### Population and data collection

We conducted a retrospective cohort study comprising women who had answered the items in the Swedish Pregnancy Planning Study on pregnancy planning [6]. From September 2012 through July 2013, women from ten of Sweden’s 21 counties were asked to participate and complete a questionnaire when registering at the Antenatal Care Unit (ACU), typically in the first trimester of pregnancy. The questionnaire comprised 148 items covering social, medical, and obstetric history, as well as questions on pregnancy planning.

Detailed information on the study population including women not approached and women who declined participation and differences in background characteristics between groups is presented by Stern et al. and by Carlander et al. [6, 30]. We present background and health characteristics found in our final study population in Table 1.

**Table 1:** Background and health characteristics of women with unplanned (*n* = 825) or planned (*n* = 1885) pregnancy presented in numbers and valid percentages

	Unplanned		Planned		Total		*P*
	n	%	n	%	n	%	
**Maternal age (years)**							
< 21	45	6	36	2	81	3	< 0.01
21–25	202	25	324	18	526	20	< 0.01
26–35	453	57	1280	70	1733	66	< 0.01
> 35	98	12	188	10	286	11	0.15
**Education (years)**							
< 9	85	10	85	5	170	6	< 0.01
9–12	362	45	674	36	1036	39	< 0.01
> 12	363	45	1091	59	1454	55	< 0.01
**BMI (kg/m** ^**2**^ **)**							
< 18.5	17	2	27	2	44	2	0.25
18.5–24.9	434	55	1041	58	1475	57	0.19
25–29.9	205	26	460	26	665	26	0.81
≥ 30	115	15	233	13	348	14	0.26
**Partner at first visit to ACU** ^**†**^	792	96	1875	99.7	2667	99	< 0.01
**Smoking 3 months prior to pregnancy**	240	29	306	16	546	20	< 0.01
**Smoking at first visit to ACU**	69	8	46	2	115	4	< 0.01
**High alcohol use 3 months before pregnancy** ^**‡**^	51	8	51	3	102	5	< 0.01
**Any alcohol use during pregnancy**	508	62	937	50	1445	54	< 0.01
**Nulliparous**	386	47	833	44	1219	45	0.22
**Number of visits to ACU**							
0–7	204	25	442	23	646	24	0.49
≥8	621	75	1443	77	2064	76	0.49
**Late detection of pregnancy** ^**§**^	25	3	10	0.5	35	1.3	< 0.01
**History of psychiatric illness**	103	12	157	8	260	10	< 0.01
**History of somatic illness**	300	36	664	35	964	36	0.57
**Foreign maternal origin**	116	14	186	10	302	11	< 0.01
**Use of folic acid 1 month before pregnancy**	71	9	812	43	883	33	< 0.01

^†^ Living with partner when filling out the questionnaire

^‡^ High alcohol use was defined as > 5 standard glasses of alcohol/week

^§^ Late detection of pregnancy defined as gestational week 10 or later

BMI: Body Mass Index; ACU: Antenatal care unit

Data on pregnancy, labour, and neonatal outcomes (up to 27 days from birth) were obtained from the Swedish Medical Birth Register (MBR). We used the Swedish unique personal identification number to link information from the Swedish Pregnancy Planning Study database to the Swedish MBR. We included data from all women who agreed to participate, completed the questionnaire, and where linkage to the MBR was possible. Data for women were excluded when pregnancy intention could not be established and when information on delivery in MBR was missing. All data were de-identified before analysis.

Beginning in 1973, the Swedish MBR contains prospectively collected, high-quality data about maternal, pregnancy, and delivery characteristics on more than 98% of all births in Sweden, as registration is mandatory and carried out by health care providers [31]. Standardized forms for antenatal, obstetric, and neonatal care are used in antenatal and delivery units in Sweden. Complications during pregnancy, delivery, and the perinatal period are diagnosed according to the International Classification of Diseases, 10th edition (ICD-10) by the responsible physician upon discharge from the hospital and data are forwarded to the MBR.

### Definitions of outcomes and variables

Pregnancy planning was measured using the LMUP tool comprising six questions [4]. These questions give a numerical score of 0–12, where 0–3 is categorized as unplanned, 4–9 as ambivalent intention, and 10–12 as planned. If a woman answered less than three of the six questions, data from that woman were excluded. If a woman answered three or more questions, imputations were used to provide a final score matching the mean score of the answered questions, as specified by Barrett et al. [4]. Based on the LMUP score, data from the women were divided into two subgroups, those with planned pregnancy (≥10) and those with an unplanned pregnancy (≤9). The women with ambivalent intention to pregnancy and unplanned pregnancy in the LMUP scoring were thus merged into one group labelled as unplanned pregnancy. When dichotomising the scale into unplanned or planned pregnancies, the cut-off is recommended between nine and ten [16].

The level of education was categorized as low (≤9 years), medium (10–12 years), or high (> 12 years). Nine years is equivalent to compulsory schooling in Sweden. This variable was chosen as a proxy for socioeconomic status [32].

LBW is defined by the World Health Organization (WHO) as < 2500 g, SGA is defined by the WHO as birth weight below two standard deviations (SD) of average for the gestational age or alternatively beneath the 10th percentile of birth weight at gestational age [33, 34]. Similarly, LGA is defined as a birth weight above two SD of average for gestational age or alternatively above the 90th percentile of birth weight at gestational age. In Sweden, two SD of average is the standard way of diagnosing SGA and LGA and is the definition used in the present study.

Neonatal outcomes were: SGA, LBW, birth weight as a continuous variable, preterm birth (birth before 37 weeks + 0 days and birth before 34 weeks + 0 days) [25], low Apgar score (defined as < 7 at 5 min), LGA, post-term birth (birth at 42 weeks + 0 days or later), and the composite variable “severe adverse neonatal outcome” including perinatal death (intrauterine or neonatal death within 27 days after birth) or resuscitation measures at birth (mask ventilation, intubation, heart compression, or correction of acidosis).

## Statistics

Descriptive statistics for continuous data are presented as median or mean, and for categorical variables as numbers and proportions. Comparative statistics for proportions were calculated using a Chi-square test, or when numbers were small, a Fisher exact test. Uni-variable and multi-variable logistic regression analyses were used for binary outcome variables. In the multi-variable regression analyses, odds ratios were adjusted in two models according to potential confounders for the different outcomes identified by use of direct acyclic graphs (DAGs) (Additional files 1–7).

Model 1. When analysing severe adverse neonatal outcome, SGA, LBW, preterm birth, and low Apgar results were adjusted for maternal age, body mass index (BMI) (< 18.5 or ≥30), smoking (still smoking or quit smoking during pregnancy), alcohol use in early pregnancy, low educational level, and origin other than Swedish.

Model 2. When analysing LGA and post-term birth, results were adjusted for maternal age, BMI, low educational level and origin other than Swedish.

Both adjusted and unadjusted odds ratios (OR) with 95% confidence interval (95% CI) were calculated. Statistical analyses were performed using IBM SPSS Statistics for Windows (version 24). Statistical significance was set at *P* < 0.05.

Supplementary logistic regression analyses were made for all neonatal outcomes in relation to pregnancy planning as a continuous variable, using the whole LMUP scale, due to the possible loss of information when dichotomising pregnancy planning. These analyses were complemented with multivariable analysis, using the same logistic regression models, when univariable analysis was found significant (Table 2).

**Table 2:** Neonatal outcomes in relation to pregnancy planning as a continuous variable

	Total (*n* = 2710)			OR	95% CI	aOR^†^	a95% CI
	n	%	Missing (n)				
**Severe adverse neonatal outcome** ^**‡**^	70	2.5	0	1.08	0.96–1.22		
**Small for gestational age**	62	2.3	60	0.87	0.79–0.96	0.89	0.79–0.99
**Low birth weight < 2500 g**	90	3.3	20	0.97	0.88–1.06		
**Preterm birth < 37 weeks + 0 days**	119	4.4	0	1.04	0.95–1.13		
**Preterm birth < 34 weeks + 0 days**	36	1.3	0	0.94	0.82–1.08		
**Apgar < 7 at 5 min**	29	1.1	22	1.02	0.87–1.2		
**Large for gestational age**	99	3.7	60	1.04	0.95–1.15		
**Post-term birth** ^**§**^	172	6.3	0	0.96	0.9–1.03		

†Model 1 (Severe adverse neonatal outcome, SGA, LBW, preterm birth and low Apgar): Adjusted for maternal age, BMI < 18,5 or ≥ 30, smoking, alcohol use, low educational level and origin other than Swedish.

Model 2 (LGA and post-term birth): Adjusted for maternal age, BMI, low educational level and origin other than Swedish.

‡Defined as intrauterine fetal death, neonatal death within 27 days of birth, or one of the following: mask ventilation, intubation, heart compressions, or correction of acidosis.

§≥42 weeks + 0 days.

## Results

### Population

**Fig. 1:**
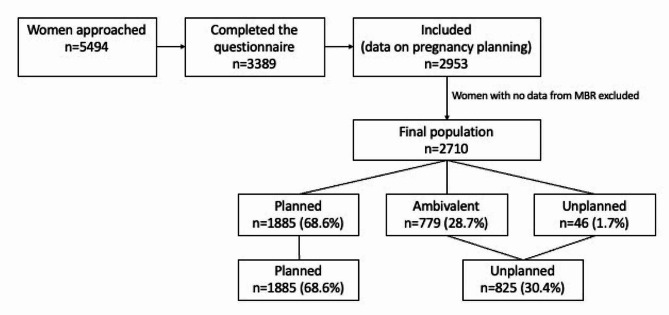
Flowchart of the study population

The study population comprised 2953 women. After excluding those who did not have data on delivery from MBR (identified by lack of a reported gestational week of delivery), 2710 women remained. Of these women 68.6% (*n* = 1885) had planned pregnancies, 28.7% (*n* = 779) had an ambivalent intention, and 1.7% (*n* = 46) had unplanned pregnancies (Fig. 1). The combined group named unplanned pregnancy, including ambivalent and unplanned pregnancies comprised 30.4% (*n* = 825) of the women.

### Neonatal outcomes

Compared with planned pregnancy, unplanned pregnancy was not associated with an increased risk for the composite variable “severe adverse neonatal outcome” (Table 3). Women with an unplanned pregnancy were more likely to give birth to SGA neonates than those with a planned pregnancy (3.6% versus 1.7%; OR 2.2, 95% CI 1.3–3.6). The difference remained significant in the multi-variable analysis (aOR 2.1, 95% CI 1.2–3.7). Correspondingly, the odds for giving birth to an SGA neonate were lower when pregnancy intention was more planned as indicated by higher scores on LMUP used as a continuous variable (Table 2).

**Table 3:** Neonatal outcomes in planned and unplanned pregnancies presented as numbers and percentages

	Total (*n* = 2710)			Unplanned (*n* = 825)			Planned (*n* = 1885)			OR	95% CI	aOR^†^	a95% CI
	n	%	Missing (n)	**n**	%	Missing (n)	**n**	%	Missing (n)				
**Severe adverse neonatal outcome** ^**‡**^ **(** ***n*** ** = 2710)**	70	2.5	0	18	2.2	0	52	2.8	0	0.8	0.5–1.4	0.7	0.4–1.3
**Small for gestational age (** ***n*** ** = 2650)**	62	2.3	60	30	3.6	14	32	1.7	46	2.2	1.3–3.6	2.1	1.2–3.7
**Low birth weight < 2500 g (** ***n*** ** = 2690)**	90	3.3	20	30	3.7	7	60	3.2	13	1.2	0.7–1.8	1.0	0.6–1.8
**Preterm birth < 37 weeks + 0 days (** ***n*** ** = 2710)**	119	4.4	0	33	4	0	86	4.6	0	0.9	0.6–1.3	0.7	0.5–1.2
**Preterm birth < 34 weeks + 0 days (** ***n*** ** = 2710)**	36	1.3	0	12	1.5	0	24	1.3	0	1.1	0.6–2.3	1.0	0.5–2.6
**Apgar < 7 at 5 min (** ***n*** ** = 2688)**	29	1.1	22	10	1.2	4	19	1.0	18	0.8	0.4–1.8	0.8	0.3–1.8
**Large for gestational age (** ***n*** ** = 2650)**	99	3.7	60	27	3.3	14	72	3.9	46	0.8	0.5–1.3	0.9	0.6–1.5
**Post-term birth** ^**§**^ **(** ***n*** ** = 2710)**	172	6.3	0	64	7.8	0	108	5.7	0	1.4	1.0–1.9	1.4	1.0–1.9

†Model 1 (Severe adverse neonatal outcome, SGA, LBW, preterm birth and low Apgar): Adjusted for maternal age, BMI < 18,5 or ≥ 30, smoking, alcohol use, low educational level and origin other than Swedish.

Model 2 (LGA and post-term birth): Adjusted for maternal age, BMI, low educational level and origin other than Swedish.

‡Defined as intrauterine fetal death, neonatal death within 27 days of birth, or one of the following: mask ventilation, intubation, heart compressions, or correction of acidosis.

§≥42 weeks + 0 days.

No significant differences were found between the groups concerning preterm birth, LGA, LBW, low Apgar score, or birth weight. Mean birth weight was 3,566 g (SD 576) in the planned group and 3,530 g (SD 590) in the unplanned group.

Neonates born to women with unplanned pregnancy were more likely to be born post-term (OR 1.4, 95% CI 1.0–1.9) than to those with planned pregnancy, and the difference remained significant in the multi-variable analysis (aOR 1.4, 95% CI 1.0–1.9) (Table 3). The odds for post-term birth were not significantly altered when related to pregnancy planning intention estimated by LMUP as a continuous variable (Table 2).

## Discussion

The main result of this study was that women with unplanned, compared to planned, pregnancies had more than double the odds for giving birth to an SGA neonate. The risk remained after considering confounders such as smoking. This result was substantiated in supplementary analysis using LMUP as a continuous variable. We also found that women with unplanned pregnancies were more likely to give birth in gestational week 42 or later. We found no other significant associations between unplanned pregnancy and neonatal outcomes.

There is a lack of studies based on the same definition of pregnancy planning as in our study when analysing SGA as an outcome variable for comparison. In three studies from the U.S. from 2015, 2022 and 2023 no associations between unplanned pregnancy and SGA nor preterm birth were seen [3, 22, 23]. Two of these studies, by Hobby et al. and Mark et al., used inverse propensity weighing to adjust for potential confounders, and they conclude that socio-economic disadvantage might be more consequential to neonatal outcome than pregnancy intention [22, 23]. None of these studies used LMUP to measure pregnancy planning and comparison is difficult given the differences in methodology and the differences between the U.S and Sweden regarding sociodemographic factors and healthcare systems.

Consistent with previous research, unplanned pregnancy was not associated with preterm birth in the present study. In a study from Belgium that included 517 women for whom pregnancy planning was assessed by use of the LMUP in the first 5 days postpartum, there were no differences in preterm birth or LBW between planned and unplanned pregnancies [9]. In a high-income country, there seems to be no increased risk for preterm birth in those with unplanned pregnancies. Conversely, in this study, women with unplanned pregnancies had higher odds for giving birth post-term, compared to women with planned pregnancies. No other studies describing this association have been found. A possible explanation might be incorrect dating of the pregnancy. This is speculative and no confident conclusion can be made. However, background characteristics of the group of women with unplanned pregnancies are associated with discrepancies between dating methods implying a possibility of misclassification of gestational age [35].

In the present study, the majority of the women with pregnancy detection after 10 weeks or later had an unplanned pregnancy. Women with unplanned pregnancies were less likely to recognize their pregnancy within the first 8 weeks, which might make them less likely to receive early prenatal care and follow early pregnancy recommendations [36, 37]. In the present study, the women with an unplanned pregnancy more often reported smoking before and in early pregnancy, high pre-pregnancy alcohol intake, and less folic acid use. The WHO recommends the first visit to the ACU in the first trimester and a minimum of eight visits to prevent perinatal mortality and LBW. Late detection could lead to less or no antenatal care and might be a factor contributing to an adverse outcome in unplanned pregnancies [38]. However, in the present study, women with an unplanned pregnancy made the same number of antenatal care visits as women with a planned pregnancy.

There was a low proportion of unplanned pregnancies and adverse neonatal outcomes in the present study, compared with global proportions [14], which may reflect the generally high quality of health care in Sweden. We note that Sweden has the highest number of legal abortions in Europe, and the use of contraception is both socially accepted and easily accessible, contributing to the low prevalence of continued unplanned pregnancies [39]. Sweden also has free maternal health care, and most pregnant women receive high-quality care during their entire pregnancy. These factors need to be considered when comparing our findings to those from studies in countries with different health care settings. Unplanned pregnancies resulting in birth in Sweden are to a large extent pregnancies where women choose to continue the pregnancy and enter antenatal care program.

### Strengths and limitations

A major strength of the present study was that the intention for pregnancy was assessed in close connection to the first visit to the ACU, usually in the first trimester, which reduces the risk of recall bias. To our knowledge, this is one of the first studies from a high-income country to use the validated LMUP tool to study neonatal outcomes. Another strength was that pregnant women in various counties, ranging from rural to urban, were included, generating a sample representative of the Swedish pregnant population. Further, data regarding pregnancy and neonatal outcomes were retrieved from a high-quality health register with a low frequency of missing data and high reliability.

This study had power limitations due to the low incidence of adverse outcomes in relation to the number of women included. Pregnancy planning, lifestyle behaviours, and socio-economic status were self-reported, which poses a risk of social desirability bias [40]. When LMUP is the studied outcome, it should preferably be used as a continuous variable and analysed using linear regression [16]. In the present study, pregnancy planning was the exposure variable and was dichotomised into well-defined groups as this was considered to be of greater clinical value. However, as dichotomising will imply loss of statistical power, the analyses were also repeated with LMUP as a continuous variable in the same logistic regression models. A possible limitation could be an incorrect estimation of gestational age, and a late detected pregnancy with postponed pregnancy dating by ultrasound could lead to either over- or under-estimation of the actual gestational age [35]. An incorrect gestational dating might affect results when studying both preterm birth, SGA, and post-term birth [41], but the likelihood is reduced in the present study as there were relatively few pregnancies detected late. Another limitation is the lack of data on women whose pregnancy was terminated, intentionally or unintentionally, after enrolment to the ACU. Also, the generalizability of this study is limited to countries with similar general healthcare and abortion services.

## Conclusions

In a Swedish setting, an unplanned pregnancy carried to birth may increase the odds for giving birth to a small for gestational age neonate. This study was not designed to examine neither causal pathways nor to assess how to decrease the risk of adverse outcome in unplanned pregnancies. The lack of other associations between unplanned pregnancy and preterm birth or other adverse neonatal outcomes suggests that women with an unplanned pregnancy in Sweden receive adequate antenatal care.

### Electronic supplementary material

Below is the link to the electronic supplementary material.

Supplementary Material 1

Supplementary Material 2

Supplementary Material 3

Supplementary Material 4

Supplementary Material 5

Supplementary Material 6

Supplementary Material 7

## Data Availability

The data that support the findings of this study are available upon reasonable request from the corresponding author. The data are not publicly available due to privacy and ethical restrictions.

## Abbreviations

LMUP: London Measure of Unplanned Pregnancy
LBW: Low birth weight
SGA: Small for gestational age
LGA: Large for gestational age
ACU: Antenatal care unit
WHO: World Health Organization
SD: Standard deviations
MBR: Medical Birth Register
DAG: Direct acyclic graphs
