# Supplementary figures and images for: Pregnancy planning and neonatal outcome - a retrospective cohort study
Source: BMC Pregnancy Childbirth. 2024 Mar 16;24:205. doi: 10.1186/s12884-024-06401-6 (PMC10944595; doi:10.1186/s12884-024-06401-6)

## DAG of outcome variable "severe adverse neonatal outcome"

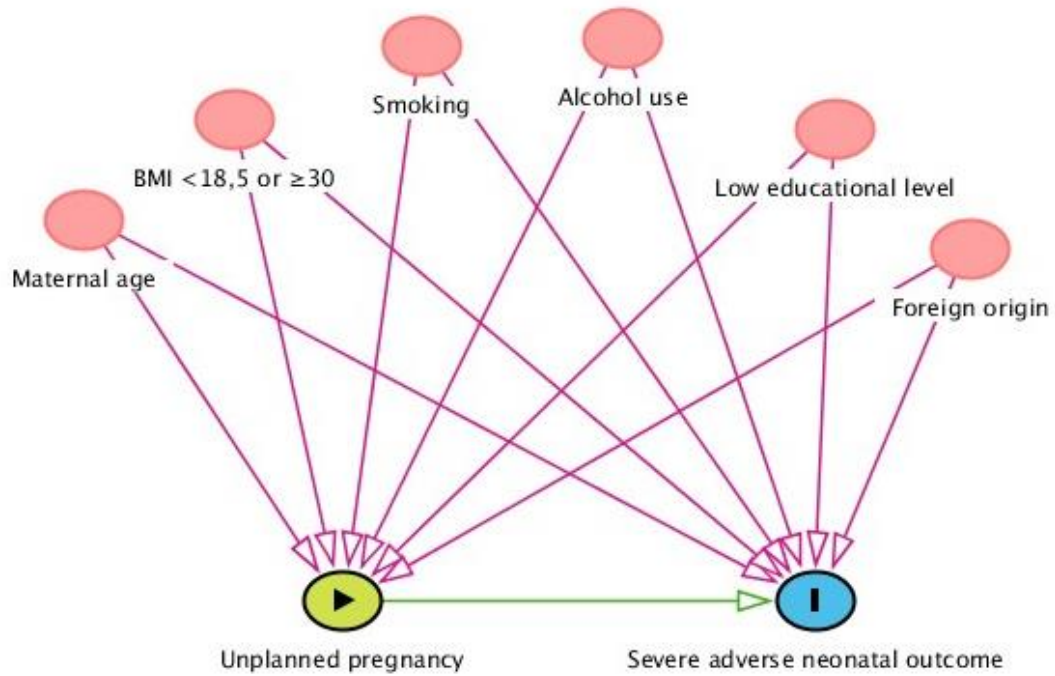

Supplement: Supplementary file 1 — Supplementary Material 1 [file 12884_2024_6401_MOESM1_ESM.pdf]

## DAG of outcome variable "small for gestational age"

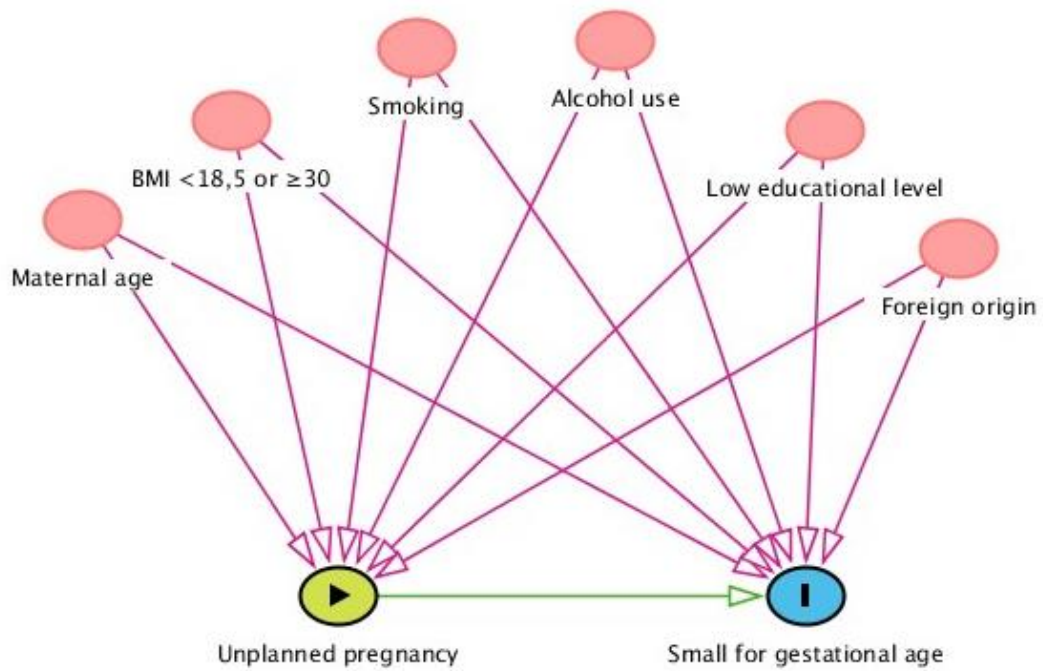

Supplement: Supplementary file 2 — Supplementary Material 2 [file 12884_2024_6401_MOESM2_ESM.pdf]

## DAG of outcome variable "low birth weight"

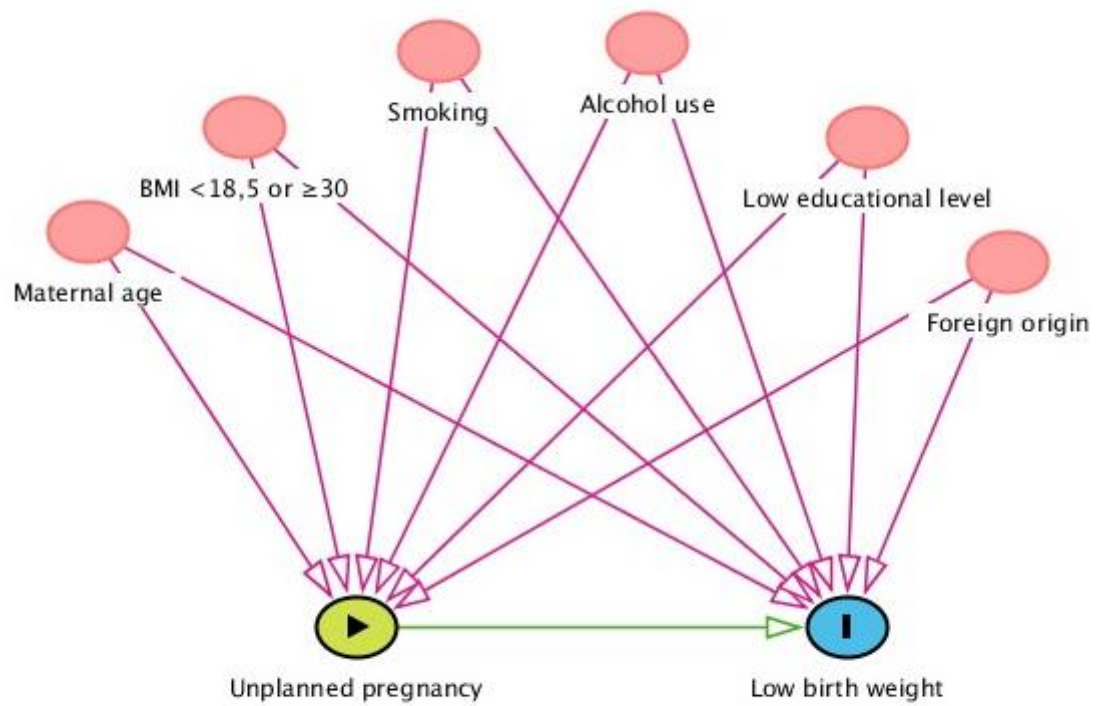

Supplement: Supplementary file 3 — Supplementary Material 3 [file 12884_2024_6401_MOESM3_ESM.pdf]

## DAG of outcome variable "preterm birth"

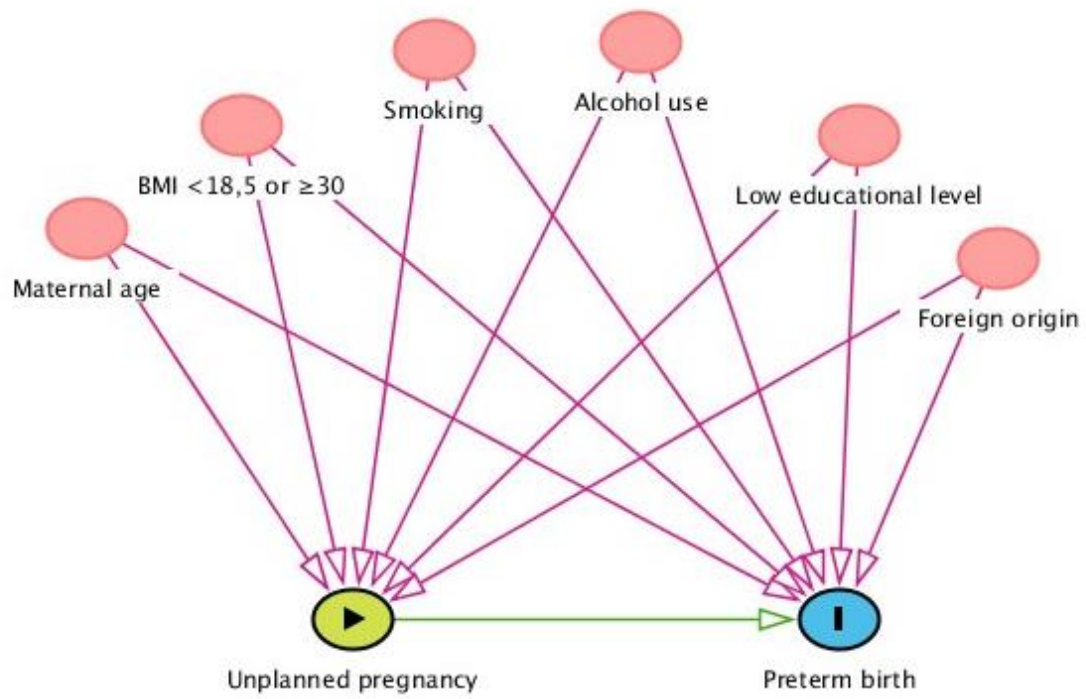

Supplement: Supplementary file 4 — Supplementary Material 4 [file 12884_2024_6401_MOESM4_ESM.pdf]

## DAG of outcome variable "low Apgar"

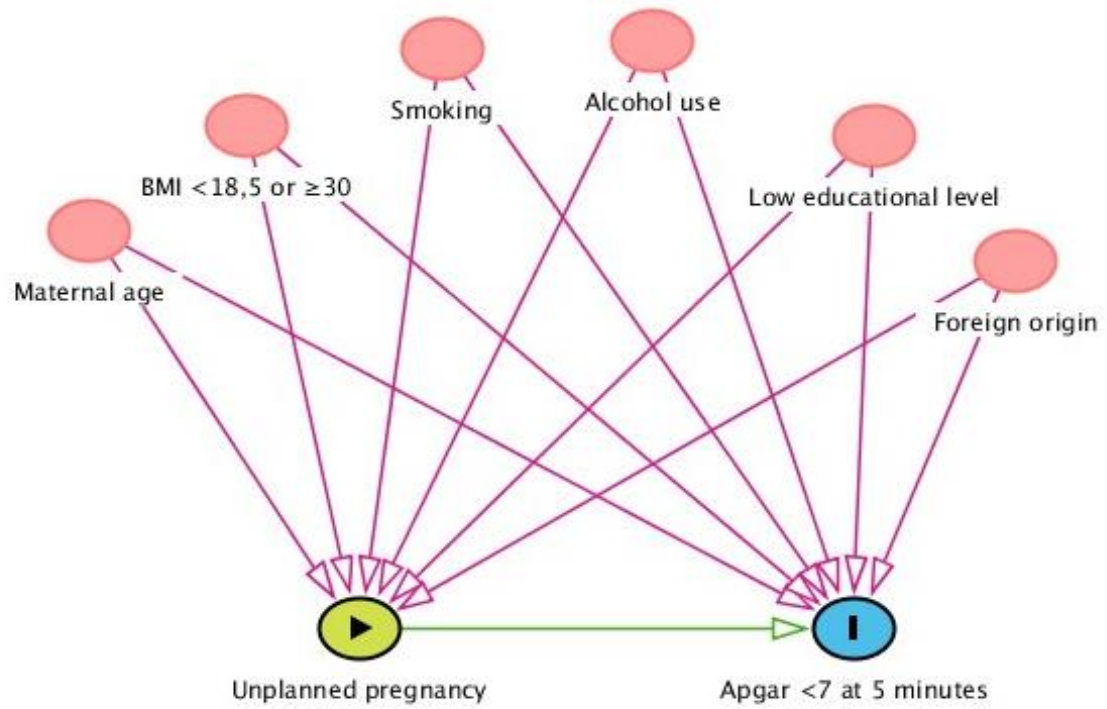

Supplement: Supplementary file 5 — Supplementary Material 5 [file 12884_2024_6401_MOESM5_ESM.pdf]

# DAG of outcome variable "large for gestational age"

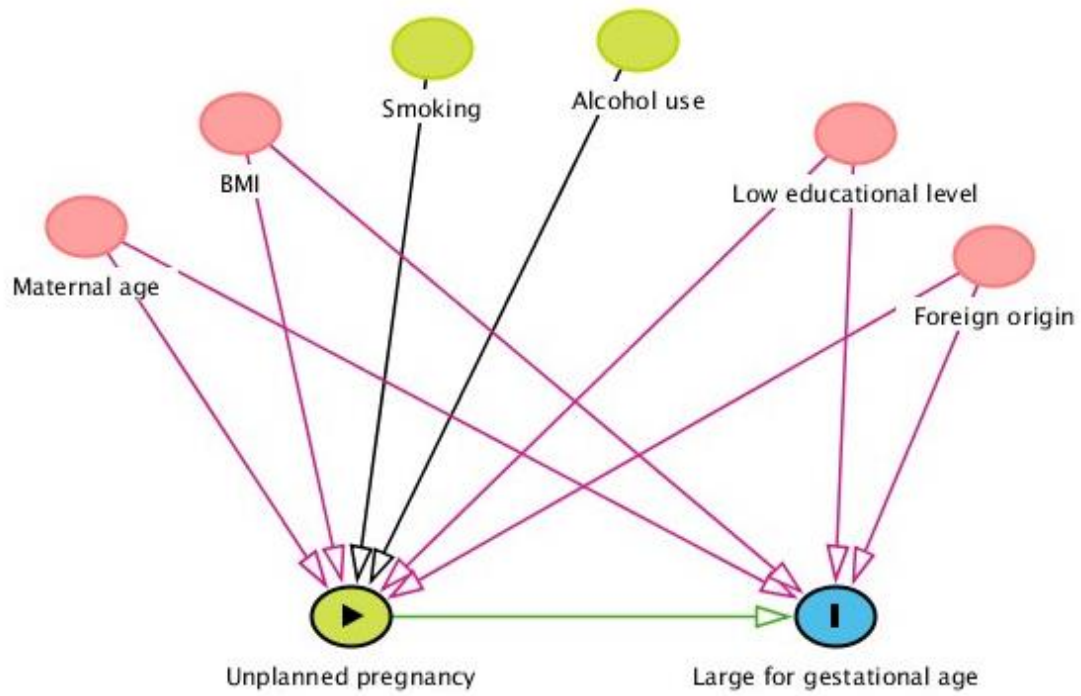

Supplement: Supplementary file 6 — Supplementary Material 6 [file 12884_2024_6401_MOESM6_ESM.pdf]

## DAG of outcome variable "post-term birth"

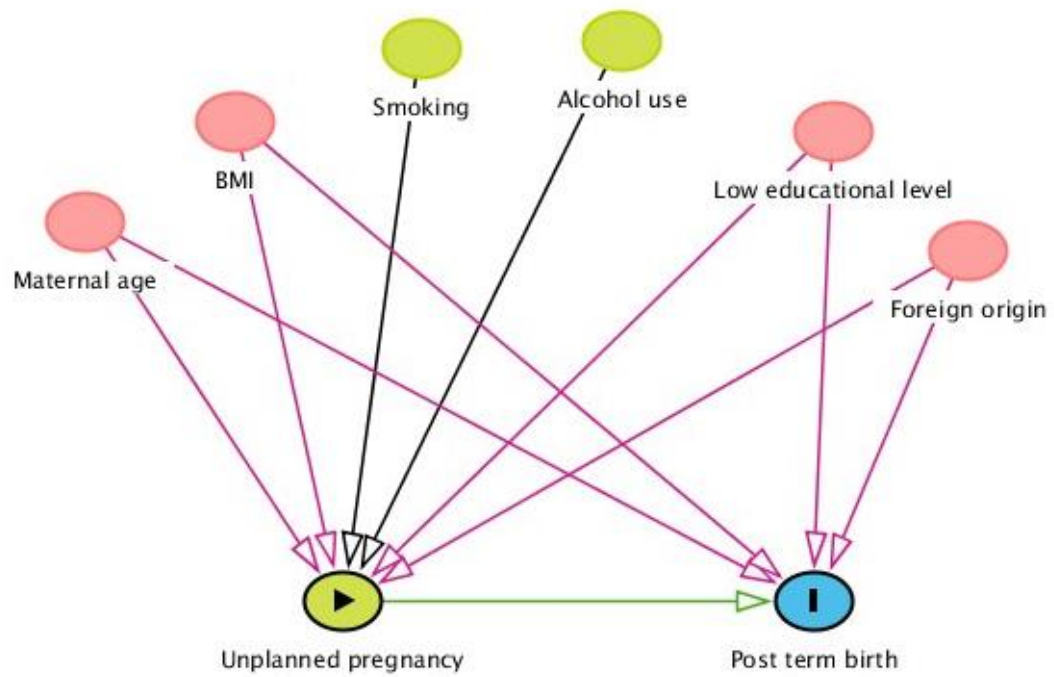

Supplement: Supplementary file 7 — Supplementary Material 7 [file 12884_2024_6401_MOESM7_ESM.pdf]
